# Eliciting the public preferences for pharmaceutical subsidy in Iran: a discrete choice experiment study

**DOI:** 10.1186/s40545-021-00345-4

**Published:** 2021-07-13

**Authors:** Mansoor Delpasand, Alireza Olyaaeemanesh, Ebrahim Jaafaripooyan, Akbar Abdollahiasl, Majid Davari, Ali Kazemi Karyani

**Affiliations:** 1 Department of Health Management and Economics, School of Public Health, Tehran University of Medical Sciences, Tehran, Iran; 2 Health Equity Research Center & National Institute for Health Research, Tehran University of Medical Sciences, Tehran, Iran; 3 Pharmacoeconomics and Pharmaceutical Administration, Tehran University of Medical Sciences, Tehran, Iran; 4 Research Center for Environmental Determinants of Health, Kermanshah University of Medical Sciences, Kermanshah, Iran

**Keywords:** Resource allocation, Subsidy, Medicine, Preferences, Discrete Choice Experiment, Iran

## Abstract

**Background:**

Deciding on pharmaceutical subsidy is regarded as a challenging issue for healthcare policymakers in Iran in most times. Public preferences, rarely attended in Iran, could be invaluable for including a particular drug in the list of subsidized medications.

**Objectives:**

The current study aims to elicit the public preferences to develop an evidence-based decision-making framework for entering a drug into the list of subsidies in Iran.

**Methods:**

Discrete Choice Experiment (DCE) was employed to elicit the public preferences. Around 34 attributes were identified based on the systematic review and interview with 51 experts. By holding an expert panel, 7 attributes were finalized, namely: the survival after treatment, quality of life after treatment (QoL), alternative treatment, age group of the target population, cost burden for the government, disease severity, and drug manufacturer country. Next, 1224 households were selected for the survey in the city of Tehran, using random cluster sampling. Data were analyzed using conditional logit model.

**Results:**

The survival after treatment (*β* = 1.245; SE = 0.053) and cost burden for the government (*β* = − 0.140; SE = 0.050) had the highest and lowest priority, respectively, in the preferences for allocating subsidy to a drug. In developed region, unlike the other two regions, the level of domestic drug production (*β* =− 0.302; SE = 0.073) was inversely associated with preferences toward allocating subsidy to a drug. In contrast to other districts, those living in district number one (*β* = 2.053; SE = 0.138) gave the highest value to promoting the QoL after treatment.

**Conclusions:**

It is suggested that policymakers pay more attention to attributes such as effectiveness and alternative treatment when developing an evidence-based framework for entering a drug into the list of subsidies. This study highlighted the public belief in the government’s subsidy for medicines, provided that, this results in an increased survival and QoL.

**Supplementary Information:**

The online version contains supplementary material available at 10.1186/s40545-021-00345-4.

## Background

Resources allocation has been always a challenging issue, with several factors, for health policymakers [1]. Health systems and healthcare organizations are faced with multiple resource constraints and are not equipped with the necessary resources to address multiple challenges at the same time [2, 3]. Some have argued that despite allocating relatively large financial resources to health sectors, the available resources are not sufficient [4, 5]. Hence, both developed and developing countries are focused on optimizing the allocation of resources [6].

The rapid growth of healthcare expenditures has become a great concern for governments and societies worldwide [7, 8]. Based on the World Bank reports, pharmaceutical expenditures represent 20 to 50% of the total health expenditures of developing countries [9]. Pharmaceutical expenditures are often the first or second contributor of direct out-of-pocket (OOP) payments and are considered as the main cause of catastrophic health payments, which cause impoverishment of middle and lower economic classes with severe or chronic disease [10]. A study conducted in 51 different countries mentioned pharmaceutical expenditures as the main cause of financial hardship, more than inpatient and outpatient expenditures [11].

Similar to other countries, the Iranian healthcare system is also faced with similar problems, particularly soaring health expenditures. Pharmaceutical expenditures are the major contributor to increased health expenditures [12]. In Iran, the pharmaceutical expenditures encompass the costs paid by both insurers and patients as out-of-pocket payments to pharmacies for those drugs that are out of the local essential drug list on the Iran Dugs List (IDL).

In Iran, special and incurable diseases receive a considerable proportion of healthcare resources due to their chronic nature, lack of definitive treatment, and high costs of treatments [13]. In recent years, the increased demand of physicians and patients for specialty medicines and expensive drugs has enhanced the total health expenditures in Iran [14].

Studies showed that pharmaceutical expenditures represented 67% (equivalent to 10,000 US dollars) of the average annual care cost of a hemophilia patient in 2014 and 62% (50,264 US dollars) of the average annual care cost of a thalassemia patient in 2015 in Iran [15, 16]. In 2018, the average annual costs per capita paid by the Iran Health Insurance Organization (IHIO) for medicines and treatment of multiple sclerosis (MS) patients was about 73 million IRR (1740 US dollar), kidney transplantation (74 million IRR ≈ 1760 US dollar), thalassemia (50 million IRR ≈ 1190 US dollar), dialysis (270 million IRR ≈ 6430 US dollar), and hemophilia (290 million IRR ≈ 6905 US dollar). Accordingly, hemophilia patients have the highest per capita cost among IHIO insures, mostly due to the high costs of drugs and importing blood factors from other countries [17].

Target 17 of the eighth goal of the Millennium Development Goals (MDGs) seeks to provide access to affordable essential medicines in developing countries [18]. There are significant inequalities in access to medicines all around the world, especially in countries with limited financial resources. Inadequate public spending, the lack of or inadequate health insurance coverage and high OOP expenditures are among the main reasons for inequality in access to medicines, according to a 2010 World Health Organization report [19].

According to the latest available data, although 70% of outpatient expenditures and 90% of inpatient expenditures are covering by health insurance funds, but the OOP was higher than 50% [14, 20]. In response to the high proportion of pharmaceutical expenditures, since 2013, Iranian healthcare system began to allocate subsidies to High Cost Medicines (HCMs) prescribing for special and incurable as well as chronic diseases, after signing an agreement between Iran Food and Drug Organization (IFDA) and health insurance funds [21]. However, the process of allocating subsidies for a particular drug seems to be complicated and with low transparency.

Because of resource limitation, special attention should be paid to the cost-effectiveness of drugs that are eligible to receive the subsidy, which are often expensive. However, currently some expensive medicines which are not cost-effective are receiving subsidy. Therefore, considering the challenges of the Iranian health system, particularly financial constraints, there should be an evidence-based framework for making such decisions. A critical review by MacLeod et al. [22] argued that when deciding about allocating resources, most policymakers consider efficiency and its related factors such as cost-effectiveness. On the other hand, procedural justice [23, 24] requires policymakers to pay attention to equity-related attributes when allocating public resources, an issue that is important for the society, in addition to cost-effectiveness [25]. Therefore, health policymakers should maintain a balance between economic, clinical, and ethical considerations when deciding how to allocate subsidies. Asking public opinions about these attributes and socially important factors for making such decisions is a common method for maintaining such balance [26].

Our literature review revealed that several studies [27–32] have investigated public preferences for pharmaceutical funding decisions. Eliciting the public preferences assists to secure public acceptance and trust, and legitimize the process before implementing any policy, or rules and regulations regarding to pharmaceutical subsidy [22, 33]. Given the different value and perspective on the equity concept in various societies [34], it is not clear whether the results of studies on social preferences of other countries, especially developing countries, reflect similar preferences of the Iranian society for pharmaceutical funding decisions or not.

On the other hand, decisions on pharmaceutical subsidy are always a big challenge for healthcare policymakers in Iran due to the differences in context between the pharmaceutical system in Iran and other developing countries. Pharmaceutical subsidy decisions in Iran are historically related to the Iranian healthcare system during the last three decades, and this can be attributed to the imposed sanctions, fluctuations in the exchange rate which dramatically reduce the value of the local currency [35]. Hence, policymakers exert big efforts to allocate subsidies to medicines to protect the public against the potential financial crisis. This is deemed as a special issue from which Iran suffers in a different manner from the other developing countries.

Therefore, eliciting public preferences concerning pharmaceutical subsidies would provide useful information for developing an evidence-based framework to assess the eligibility of a drug to receive the subsidy in Iran. According to the best knowledge of the authors, little is known about the preferences of the Iranian public regarding allocating resources decisions for pharmaceutical.

## Objectives

The current study aims to elicit the public preferences to develop an evidence-based decision-making framework for entering a drug into the list of subsidies in Iran.

## Methods

Conjoint analysis (CA) is a powerful method for eliciting the preferences of different stakeholders [36]. Several studies have used this method to elicit the preferences of public, patients, and policymakers for making informed decisions [32, 37, 41]. CA has several types, one of them is Discrete Choice Experiment (DCE). Although, there are limitations related to selecting the number of attributes and levels that might be included in the final design of the DCE-using study [42, 43], this method is the most widely used in health economics [44]. Therefore, in the present study, the DCE was administered to elicit the public preferences between October 2018 and **December** 2019 according to the following steps [45]: (1) Identification of attributes; (2) determination of the final attributes and levels; (3) experimental design (presenting the scenarios); (4) piloting; (5) data collection, and (6) data analysis.

### Identification of attributes

In the first step, the literature was searched systematically through the following databases: PubMed, Embase, Scopus, and Web of Science. In addition, Google and Google Scholar were searched to identify any non-indexed published articles or gray literature (Additional file 1: Table S1 and Fig. S1). After completing the systematic review, important attributes (criteria) of resource allocation of medicines were extracted and listed. The systematic review of the literature revealed a number of attributes about one hundred and twenty-four (124).

Then, the opinions of experts (key informants) were probed through semi-structured interviews, as they have been selected using purposive and snowball sampling techniques up to saturation [46, 47]. The interview guide included enquiring about the interviewees' demographic characteristics and other items related to the study objectives (Additional file 1).

Totally, 51 experts, including policymakers, who were experienced in the challenges of drug subsidy or funding were included in such interviews (Additional file 1: Table S2). After completing the interviews, important attributes related to allocating subsidies to medicines were identified. The interviews with key informants demonstrated a number of additional 64 attributes.

Eventually in this step, a list of different attributes for allocating subsidy and resources to essential drugs was compiled and developed, both in Iran and other countries.

### Determination of the final attributes and levels

In the second step, research team intended to determine the final attributes and levels of subsidy allocation to drugs. Therefore, a meeting was carried out to examine the identified attributes, in which, team members in this meeting excluded the repetitive attributes and discussed all others thoroughly aiming to reach agreement on an initial set with a reduced number. Thus, this step yielded 34 attributes (Additional file 1: Table S3).

According to the recent reviews, it has been reported that most DCEs used a number of attributes between 4 and 7 [42, 48]. Up on which, a panel comprised five experts discussed the aforementioned attributes [49, 50]. In this meeting, the panel members were asked to select, from among the 34 previous attributes, seven important attributes that policymakers need to consider in allocating subsidies for drugs in the Iranian health system (Additional file 1). Hereupon, the panel came up with a list of seven attributes, including: increasing survival after treatment, promoting quality of life (QoL) after treatment, alternative treatment, age group of the target population, cost burden for the government, disease severity, and Medicine manufacturer country (Table 1). Moreover, the appropriate number of levels for each attribute was determined (Table 1).

**Table 1:** Attributes and levels used in the DCE

Attribute	Definition	Level
		Pharmaceuticals A, B
Increasing survival after treatment	The average number of years increased to the patients' life by taking the drug	No effect on the patients' longevity (remaining in the previous lifetime)
		Low = 1 year
		Average = 5 year
		High = 10 year
Promoting quality of life after treatment	Improved health-related quality of life due to consuming a drug. According to the World Health Organization, four dimensions of health are physical, mental, social, and spiritual. Therefore, in this study, we only considered the health-related quality of life	No effect on QoL of patients (previous QoL)
		Low improvement in QoL (15%)
		Average improvement in QoL (30%)
		High improvement in QoL (50%)
Alternative treatment	Other treatments such as surgery, radiotherapy, and other drugs with similar mechanisms of action	Yes
		No
Age group of the target population	The age range of those who consume the drug	Less than 18 years of age
		18 to 60y
		Over 60y
		All age groups
Cost burden for the government	The annual budget that the government allocates as subsidy for medicines	Low = 10 million IRR (240 US dollar)
		Average = 100 million IRR (2380 US dollar)
		High = 500 million IRR (11,900 US dollar)
Disease severity	Patients' longevity and QoL before the onset of drug use	Mild = high longevity (15 years), moderate QoL [60%]
		Moderate = high longevity (15 years), low QoL (30%)
		Severe = low longevity (up to 3 month) low QoL (30%)
Drug manufacturer country	The final product is produced in Iran or is imported	Domestic production
		Imported

### Experimental design (presenting the scenarios)

As shown in Table 1, we had three attributes of four levels, two attributes of three levels, and two of two levels. Accordingly, all possible combinations of the attributes levels gave the number of scenarios equal to $4^{3} \times 3 ^{2} \times 2 ^{2} = 2304$ and $\frac{2304 \times 2303}{2} = 2653056$ choice pairs. In the third step, to produce pairs of choices with optimal number and efficiency, the D-optimal designing method was employed. This method selects the sets of choices that possess the most possible information. SAS software (version 9.4) was used to design the experiment and all of its internal macros were used as follows:

1. First, all the performances of the full factorial model were made using FACTEX procedure.
2. The optimal choices with the maximum value of D-efficiency criterion were introduced with the OPTEX procedure.

Therefore, a 21-choice set in three blocks (each with 7 items) design was introduced as an optimal design using a fractional-factorial design and the D-efficiency method.

It is noteworthy that a further dominated choice task as warm-up and internal validity test for rational trading behaviors was applied, eventually, 24-choice sets were developed in three blocks (each with 8 items). Those who gave a wrong answer to the dominant scenario were excluded from the study. Scenarios were developed using a generic approach (drug A and drug B) and each set only contained two scenarios. An example of developed scenarios is presented in Table 2.

**Table 2:** Example choice question from study

Pharmaceutical A		Pharmaceutical B
Average (5 years)	Survival	Low (1 year)
High improvement in QoL (50%)	Quality of life	Low improvement QoL (15%)
No	Alternative treatment	Yes
5 years	Age group	18 to 60 years
High: 500 million IRR (11,900 US dollar)	Cost to government	High: 500 million IRR (11,900 US dollar)
Severe: low longevity (up to 3 month); low QoL (30%)	Disease severity	Mild: high longevity (15 years), moderate QoL (60%)
Domestic production	Drug manufacturer country	Imported
Which pharmaceutical do you prefer to be subsidized? Pharmaceutical A Pharmaceutical B		

### Piloting

In the fourth step, a questionnaire was designed and piloted. The questionnaire contained two parts: (a) DCE; and (b) demographic and socioeconomic status (Additional file 2). For the first part, the hypothetical status of two drugs was presented in two scenarios, and participants were asked to choose the one they think is eligible for receiving the subsidy. To collect data, a contract was signed with the Iranian Students Polling Agency (ISPA) in 2019. The pilot study was conducted on 48 households non-randomly selected by two trained researchers.

### Data collection

In the fifth step, the sampling framework was determined. The sample size was determined according to the Cochran formula, a non-response rate of 5% (based on the pilot study), and design effect (DE) of 1.5, which yielded a sample size of 1224 households. Participants were selected using the random cluster sampling technique so that 22 districts of Tehran were divided into three categories of highly developed, semi-developed, and less-developed. Then, from each category, two districts were randomly selected and some blocks were randomly selected.

Sampling technique was designed based on differences between the 22 districts in Tehran city concerning the geographical distribution, and considering the economic, social, spatial, cultural, and recreational indicators [51] (Additional file 1). After determining the sample size and choosing the sampling technique, a meeting was held to brief the questioners of the ISPA organization. They were asked to refer to households living in districts number 1, 6, 4, 11, 14, and 17. They were mandated to interview the head of family or a family member with at least 18 years of age and at least 12-year formal education (basic education). Interviews, on average, took about 20 min. Based on the findings of the pilot study, the inclusion attribute was having a minimum education level of Diploma.

### Data analysis

In the sixth step (final step), after completing all questionnaires, the collected data were entered into SPSS software version 22. In total data of 1224 households living in the city of Tehran were collected. Fourteen questionnaires were excluded due to incompleteness and 164 were excluded due to no selecting the dominant option. Hence, 1046 questionnaires were confirmed. Descriptive statistics were used to analyze demographic and socioeconomic information, and the conditional logit model was used for DCE.

Random utility framework is the basis of DCE data analysis, in which the respondent can imagine another option in a choice set that maximizes their utility. One of the most popular models of DCE data analysis, which is available in almost all softwares, is the conditional logit model. This regression model considers the discrete structure of choices as a dependent variable and examines the relationship between the probability of selecting a choice and attributes. The conditional logit model is often employed when the variables affecting the choice of people change during the selection of options. The β coefficients indicate the level of utility related to the changes in the level of attributes compared to the reference level. The non-significance of β coefficients associated to the attributes in the conditional model does not mean that the attributes is ineffective in the decision of individuals. Rather, the specified attributes with the intended levels have not been considered by the respondents [44].

In the conditional logit regression, various criteria are used for the goodness of fit, the most important of which are likelihood ratio (LR) and log likelihood. LR indicates the overall significance of the regression model, and its high value indicates the reliability of the model. When the value of log-likelihood statistic is closer to zero, the model seems to be more appropriate. Pseudo *R*^2^ is a measure that shows the fitting power of the model. In logit models, pseudo *R*^2^-statistics are used to evaluate the goodness of model fit instead of *R*^2^. The value of pseudo-*R*^2^ statistics in the range of 0.2 to 0.3 is equivalent to the value of 0.7 to 0.9 of *R*^2^ in other regression models. Generally, when pseudo *R*^2^ value is greater than 0.2, this indicates a good fit [52].

To determine the importance of each attribute, relative importance is used as a scale for the respondent. In fact, this scale is calculated based on the partial change that occurs in the in log-likelihood of model after adding the attribute to the model which compared to the other attributes [53]. Data were analyzed using SPSS version 22 and STATA version 14.2. Statistical significance was considered when *p*-value < 0.05.

### Ethical considerations

All ethical considerations were observed in the present study. Before interviewing with households, the objectives of the study were explained to them and, if agreeing, informed written consent was obtained. Participants were ensured about the confidentiality of information. Before referring to households, all necessary permissions were obtained. The current study is approved by the ethics committee of the Tehran University of Medical Sciences (code: IR.TUMS.SPH.REC.1395.1786).

## Results

In this part, we discussed the findings. Initially, the demographic and socioeconomic characteristics of the participants are discussed. Then, the findings of logistic regression are provided. Finally, the results of regression models of the effect of demographic and socioeconomic characteristics on the preferences of participants are described.

Of 1224 questionnaires, 1046 were eligible for analysis for a return rate of 90%. Participants included 455 males and 591 females. Most of the participants were female (56.5%) and married (74.5%). The mean age of participants was 55.29 ± 13.785. The youngest and oldest participants were 19 and 80 years old, respectively. For 61.2% of households, the average monthly income was lower than 41 million IRR ≈ 976 US dollar. Besides, 13.3% of households had a history of hospitalization during the past year, and 55.7% of them reported consuming a prescribed drug during the past year. The socioeconomic characteristics of households are described in Table 3.

**Table 3:** Demographic and socioeconomic characteristics of households (2019)

Variable			Frequency	Percentage	
Gender	Male		455	43.5	
	Female		591	56.5	
Age	Less than 41 years of age		201	19.3	
	41–60 years		419	40	
	Older than 60		426	40.7	
Marriage status	Married		779	74.5	
	Non-married (single, divorced)		267	25.5	
Head of household	Yes		418	40	
	No		628	60	
Family members	Less than three		3178	21.7	
	Three		4508	30.8	
	More than three		6958	47.5	
Education level	Diploma (high school)		530	50.7	
	Associates degree—Bachelorette		390	37.3	
	M.Sc.—Ph.D.		126	12	
Employment status	Public sector		85	8.1	
	Private sector		344	32.9	
	Retired		136	13	
	Unemployed (unemployed, housewife, student, duty soldier)		481	46	
Basic health insurance	No health insurance		140	13.4	
	Social security health insurance coverage		659	63	
	Other		247	23.6	
Complementary health insurance	Yes		400	38.2	
	No		646	61.8	
Suffering chronic diseases (head of household)	Yes		91	8.7	
	No		955	91.3	
Suffering chronic diseases (family member)	Yes		114	10.9	
	No		932	89.1	
Level of development of districts of Tehran	Highly developed	District 1	199	19	37.7
		District 6	195	18.7	
	Semi-developed	District 4	173	16.5	34.9
		District 11	192	18.4	
	Less-developed	District 14	130	12.4	27.4
		District 17	157	15	
Average monthly income of household (IRR or US dollar)	Less than 20 million IRR (465 US dollar)		159	15.2	
	20 to 40 million IRR (930 US dollar)		481	46	
	More than 40 million IRR (930 US dollar)		406	38.8	
Total			1046	100	

In the second part, the results of the conditional logit model were discussed. The results indicated that the highest utility for selecting a drug was for drugs with high (10 years; *β* = 1.245; SE = 0.053) and medium (5 years; *β* = 0.878; SE = 0.05) levels of survival, followed by high (50%; *β* = 0.862; SE = 0.047) and medium (30%; *β* = 0.668; SE = 0.053) levels of QoL. Then, lack of alternative treatments (*β* = 0.451; SE = 0.029), low increase in QoL (15%; *β* = 0.447; SE = 0.047), and all age groups (*β* = 0.273; SE = 0.050) were other important levels. The lowest utility rate was devoted to the low increase in survival rate (one year; *β* = 0.156; SE = 0.051). Expenditure of 500 million IRR ≈ 11,900 US dollar, severe disease, and a target population of older than 60 years were the most important levels which were caused under utility. A target population older than 60 years obtained the highest negative value (*β* =− 0.477; SE = 0.047). In the present study, the coefficient (SE) for domestic production was—0.061 (0.045), but it was not statistically significant (*p* > 0.05). This indicates that domestic drugs are less likely to receive the subsidy, but its effect on the utility of participants was not significant (*p* > 0.05).

Increasing survival after treatment was the most important attribute in the present study (34%), followed by promoting QoL (26.5%), alternative treatment, and, age group of the target population. On the other hand, cost burden for the government and disease severity obtained the lowest importance. Medicine manufacturer country did not have a significant effect on preferences toward entering a drug into the list of subsidized drugs. Pseudo-*R*^2^ was 0.157, and it showed good fit of our model (Table 4).

**Table 4:** Results for conditional logit model for eliciting the public preferences for pharmaceutical subsidy in Iran

Attribute	Level	β (SE)	*P*-value	Relative importance (percentage)
Survival	Low (one year)	0.156 (0.051)	< 0.05	34.1
	Average (5 years)	0.878 (0.051)	< 0.05	
	High (10 years)	1.245 (0.053)	< 0.05	
Quality of life	Low (15%)	0.447 (0.047)	< 0.05	26.6
	Average (30%)	0.668 (0.053)	< 0.05	
	High (50%)	0.862 (0.047)	< 0.05	
Alternative treatment	No	0.451 (0.029)	< 0.05	22.6
Age group	18–60 years	0.051(0.044)	0.245	7.8
	Older than 60 years	$-$ 0.477 (0.047)	< 0.05	
	All age groups	0.273 (0.050)	< 0.05	
Cost to government	Average (100 million IRR or 2380 US dollar)	0.071 (0.050)	0.160	4.1
	High (500 million IRR or 11,900 US dollar)	$-$0.140 (0.050)	< 0.05	
Disease severity	Moderate	$-$0.064 (0.038)	0.095	4.8
	Severe	$-$0.143 (0.043)	< 0.05	
Drug manufacturer country	Domestic production	$-$0.061 (0.045)	0.181	0
Goodness of fit	LR Chi^2^[15]	1601.910		
	Log likelihood	− 4274.270		
	Pseudo-*R*^2^	0.157		
Number of observations	14,644			

In this part, findings concerning the effect of variables such as gender, age, education level, and urban development on the preferences of the participants are investigated. Result of the conditional logit model demonstrated that levels of disease severity have a significant effect (*p* < 0.05) on reducing the utility of women for subsidizing a drug, in contrast to men (*β* =− 0.183; SE = 0.059). In all three age groups, increasing patient survival had the highest utility, however, its utility was higher for those older than 60 years of age. In all three age groups, the severe disease was associated with decreasing utility, however, only for those younger than 60 years of age, it was statistically significant (less than 41 years; *β* =− 0.287; SE = 0.106), (41 to 60 years; *β* =− 0.151; SE = 0.06). For those Diploma holders, levels of expenditure of 500 million IRR (11,900 US dollar) (*β* =− 0.222; SE = 0.072) and severe disease (*β* =− 0.147; SE = 0.060) were associated with decreasing utility, in contrast, for those with a university degree, its effect was not significant (*p* > 0.05).

Preferences of those living in three regions of Tehran separated by development level showed that the highest utility was for increasing the survival rate. In five districts of Tehran, the highest utility for allocating subsidy was for increasing the survival after treatment. In contrast to other districts, in district number one, the highest utility was for promoting the QoL (*β* = 2.053; SE = 0.138). In districts that are highly developed; both moderate (*β* =− 0.173; SE = 0.062) and severe (*β* =− 0.256; SE = 0.068) levels of disease severity were associated with decreasing utility. In this region, domestic production was also associated with decreasing utility, but in the other two regions, this effect was not statistically significant (*β* =− 0.302; SE = 0.073). In districts semi-developed (*β* =− 0.287; SE = 0.103) and less-developed (*β* =− 0.183; SE = 0.093), a cost burden 500 million IRR ≈ 11,900 US dollar) was associated with decreasing utility, but in highly developed districts, its effect was not statistically significant (*p* > 0.05).

## Discussion

In this study, we collected data of 1224 households in the city of Tehran to elicit their preferences in order to develop an evidence-based decision-making framework for including a drug into the subsidy list. Governments and agencies which are responsible for making decisions regarding reimbursing drugs are increasingly concerned about how to elicit society's preferences [30]. For this reason, in countries such as Australia, the Pharmaceutical Committee consists of a member of society [30]. Or, in countries such as the United States, Britain, New Zealand, the Netherlands, Israel, and Sweden, public preferences have a high weight in decisions [54]. However, in Iran, public preferences are less considered when making decisions regarding allocating subsidy to a drug, which is consistent with findings of similar studies conducted in Germany and Cyprus [55, 56]. It worth noting that because studies, which investigated the preferences toward pharmaceutical decisions, have used various attributes, caution should be taken when comparing their findings.

In the present study, participants gave the highest weight to increasing survival after treatment, promoting QoL after treatment, and alternative treatments. The first two important attributes in choosing the drug to enter the list of subsidized drugs were related to the effectiveness. Each attribute contained some levels, which some of them had a significant influence on preferences. In line with this finding, Whitty et al. in Australia [32], Polisena et al. in Canada [57], Kwon et al. in South Korea [31], Schwappach in Germany [25], and Bryan et al. in the UK [58] have reported similar results. Although in the present study, the rank of importance of attributes related to drug effectiveness was different. Whitty et al., which elicited the preferences of Australians concerning allocating resources to the pharmaceutical market of Australia using the DCE method, mentioned increasing survival after treatment as the most important attribute [32]. In the present study also the most important attribute was increasing survival after treatment, which is similar to other studies.

In a national survey, Polisena et al. examined eight important priorities in drug reimbursement decisions related to rare diseases and mentioned improving the QoL after treatment as the most important attribute. They also reported the increasing survival after treatment as the seventh important attribute [57]. The findings of the present study are somehow consistent with the study by Polisena et al. [57]. The difference concerning the importance of increasing survival after treatment can be attributed to using the DCE methodology. Also, it worth noting that in the present study we tried to elicit preferences toward all drugs, while they only investigated drugs related to rare diseases. Kwon et al. have used MCDA methodology to elicit preferences concerning drugs related to cancer treatment using eight attributes and mentioned clinical benefits (effectiveness) as the most important attribute [31].

Alternative treatment was the third important attribute in the present study, so that drugs with no alternative gained the highest importance for receiving the subsidy. The findings of the present study are consistent with results reported by Chim et al. in Australia [30] and Linley and Hughes [29] in the UK. Hence, based on the findings, drugs related to cancer treatment and rare diseases with no alternative gained higher importance to be included in the list of subsidized drugs. Also, Chim et al. [30] reported that, based on the preferences of Australians, drugs related to rare diseases did not have a high priority but public resources should be used to reduce their price [30].

In the present study, the age group of the target population, cost burden for the government, and disease severity gained the least importance. The fourth important attribute for allocating subsidy for a drug was the age group of the target population. Concerning this attribute, participants emphasized drugs which are using by all age groups. As evidence regarding the importance of age as an attribute for allocating resources is controversial, using this attribute for including a drug in the list of subsidized drugs would be challenging. Some studies mentioned age as an important attribute for allocating resources, so that children and young adults should be prioritized over the elderly [55, 56, 59–61]. On the other hand, some studies argued that age should not be used as an important attribute for allocating resources or less importance should be given to this variable [30, 62–64]. Besides, some studies on allocating resources related to drugs mentioned age as an important factor. For example, Stafinski and Menon investigated the preferences of Canadians toward allocating more resources to cancer treatments and reported that participants tended toward younger adults who suffer from cancer compared to the elderly [65]. Different studies emphasized considering certain age groups or generally not considering age as an attribute in resource allocation, which is consistent with the findings of the present study. Similar findings are reported by Dolan and Cookson, which reported the reasoning of participants for giving a similar weight to all age groups [66]. Farmakas et al. reported that there are studies that mentioned the role of nationality in prioritizing age as an attribute for allocating resources [56]. In this line, the fact that most of the Iranians are Muslim probably has played a significant role in prioritizing age as an important attribute for allocating resources for subsidizing drugs. However, further studies are needed to confirm this hypothesis.

The last attribute for entering a drug into the list of subsidized drugs was the cost burden for the government. Based on the findings, public did not tend to drugs with a cost 500 million IRR ≈ 11,900 US dollar, the declining trend initiated. The lower importance of this dimension can be attributed to the fact that a third party pays most of the pharmaceutical expenditures in Iran (that is, the government pays the bills by oil dollars, not using taxes).

Also, regarding the soared exchange rate and outrageous sanctions during the past decades, public expect the government to cover a higher proportion of pharmaceutical expenditures; however, preferences for governmental pharmaceutical spending should consider increasing survival after treatment and QoL after treatment. That is, based on the preferences of the participants, for more expensive drugs, even in cases with high effectiveness, the government should not spend substantial resources to subsidize a drug. Whitty et al. also reported that the Australians tended to set a range for government health expenditures [32] Whitty et al. also reported that the Australians tended to set a range for government health expenditures (which indicates that the public are willing to place a limit on the amount of subsidy paid by the government) [32]. It seems that, based on the preferences of public, governments should give priorities to cost-effectiveness attribute when deciding about entering a drug into the list of subsidized drugs; however, more attention should be paid to insurance policies, because due to inappropriate measures the OOP expenditure for prescribed drugs has increased. Our findings are consistent with the study by Farmakas et al., which reported that cost burden obtained lower importance for allocating resources of Cyprus health system, ranked fifth out of six [56]. However, our findings are inconsistent with Whitty et al. [32]. This difference can be attributed to the fact that the Australian health system is tax-based. Hence, it can be argued that because Australians are directly financing their health system, they are more sensitive about costs; however, in contrast, in Iran Oil dollars are the main source of financing the health system and, public are less sensitive to the costs.

The fifth important attribute for entering a drug into the list of subsidized drugs was disease severity. According to the findings, concerning this attribute, public did not tend much towards drugs for severe diseases. Preferences toward this attribute for drugs related to diseases with moderate severity were not lower than that of diseases with mild severity, while for diseases with higher severity it was different, so that a lower tendency was observed toward allocating subsidy to drugs related to these diseases. In the present study, the severe disease was defined as maximum survival of 3 months and a QoL of 30%, and moderate disease was defined as maximum survival of 15 years and a QoL of 30%. Based on the findings, the government should not provide subsidies to drugs related to severe diseases (e.g., cancer patients who have low survival and QoL); however, for diseases with moderate severity (e.g., diabetes) the situation was different. Hence, it can be argued that, public tended toward spending resources on programs that are intended to increase awareness and palliative care, instead of subsidizing drugs related to severe diseases. Most of the studies mentioned disease severity as an important factor for allocating resources [67, 68], while in the present study this attribute obtained low importance. Kwon et al. also mentioned that the disease severity obtained low importance in a sample of South Koreans relative to other studies. They also reported that effectiveness and cost-effectiveness obtained higher priorities [31]. Therefore, based on the currently available evidence, a lower weight should be given to this attribute when deciding on entering a drug into the list of subsidized drugs. Furthermore, concerning that the manufacturing country did not have a significant effect on public preferences toward subsidizing a drug, further studies should be conducted on this attribute.

The current study also had limitations, including elucidating preferences of those with a minimum education of Diploma in the city of Tehran. It worth noting that despite using cluster sampling, because we only interviewed those with a minimum education of Diploma, the impact of selection bias on the findings cannot be rejected. Also, due to the high cost and executive problems of implementing the research in all provinces, we only elicited the preferences of those living in the city of Tehran. Preferences may vary in different regions of the country. Therefore, caution should be taken when generalizing the findings to the whole country. Hence, the authors recommend performing a national wide study to elicit preferences toward entering a drug into the list of subsidized drugs.

Another limitation of the current study was that the study participants were not asked to state their preferred choices, rather, a discrete choice was made available for them to choose. They might add so many new reasons to include a given drug in the subsidy list as preference. However, due to the nature of the study they were provided discrete choices.

## Conclusion

This study provided a clear picture of the Iranian's preferences for allocating resources to drugs. Despite having limitations, this study is the first of its kind on eliciting, public preferences concerning the distributive justice of pharmaceutical resources, by face-to-face interviews.

Based on the findings, when deciding on entering a drug into the list of subsidized drugs, increasing survival after treatment, promoting QoL after treatment, and alternative treatments are important attributes that should be considered. Hence, the health policymakers of the country should consider these attributes (effectiveness and alternative treatment) for designing an evidence-based framework for entering a drug into the list of subsidized drugs. This study highlighted the public belief in the government’s subsidy for medicines, provided that, this results in an increased survival and QoL. Present study also demonstrated that in order to enter a drug into the list of subsidized drugs, attributes such as age group of the target population, cost burden for the government, and disease severity should be considered as attributes of relatively low importance.

Using these attributes, as important social values, in the country's pharmaceutical subsidy decisions will reduce conflicts between, public and policymakers, improves the alignment of government preferences with social preferences, and causes greater legitimacy of decisions [22, 33]. The current study aimed to elicit the preferences of Iranians towards allocating subsidies to drugs as well as the extent of reflecting these values in policymakers' decisions. Based on the findings further studies are needed to further examine these two issues. The findings can be generalized to other developing countries with similar contexts. However, caution should be taken when generalizing the findings.

## Supplementary Information

**Additional file 1.** Methodology of identification of attributes (literature systematic review and interview with experts).

**Additional file 2.** Questionnaire of public preferences evaluation.

## Data Availability

The data that support the findings of this study are available on reasonable request from the corresponding author MD. The data are not publicly available due to the data containing information that could compromise participant privacy.
