# Correction to: Eliciting the public preferences for pharmaceutical subsidy in Iran: a discrete choice experiment study

**DOI:** 10.1186/s40545-021-00358-z

**Published:** 2021-09-09

**Authors:** Mansoor Delpasand, Alireza Olyaaeemanesh, Ebrahim Jaafaripooyan, Akbar Abdollahiasl, Majid Davari, Ali Kazemi Karyani

**Affiliations:** 1 Department of Health Management and Economics, School of Public Health, Tehran University of Medical Sciences, Tehran, Iran; 2 Health Equity Research Center & National Institute for Health Research, Tehran University of Medical Sciences, Tehran, Iran; 3 Pharmacoeconomics and Pharmaceutical Administration, Tehran University of Medical Sciences, Tehran, Iran; 4 Research Center for Environmental Determinants of Health, Kermanshah University of Medical Sciences, Kermanshah, Iran

## Correction to: J Pharm Policy Pract (2021) 14:59 https://doi.org/10.1186/s40545-021-00345-4

Following the publication of the original article [1], the authors notified us of a few mistakes:

- In the Results section of the Abstract, “disease severity (*β* = − 0.143; SE = 0.043)” should actually read “cost burden for the government (*β* = − 0.140; SE = 0.050)”.
- In the Results section of the body of the article, the phrase “Increasing survival after treatment was the most important attribute in the present study (36%), followed by promoting QoL (27%), alternative treatment” should read “Increasing survival after treatment was the most important attribute in the present study (34%), followed by promoting QoL (26.5%), alternative treatment”.
- In the 7th paragraph of the Discussions section, the phrase “The last attribute for entering a drug into the list of subsidized drugs was disease severity” should read “The fifth important attribute for entering a drug into the list of subsidized drugs was disease severity.”.
- In the 8th paragraph of the Discussions section, the phrase “The fifth important attribute for entering a drug into the list of subsidized drugs was the cost burden for the government.” should read “The last attribute for entering a drug into the list of subsidized drugs was the cost burden for the government.”.
- In Table 4, the values for the relative importance (last column) are changed:
  - 36 becomes 34.1
  - 27 becomes 26.6
  - 17.20 becomes 22.6
  - 13.10 becomes 7.8
  - 3.40 becomes 4.1
  - 3.30 becomes 4.8

The original article has now been corrected.

## References

[1] Delpasand M, Olyaaeemanesh A, Jaafaripooyan E, Abdollahiasl A, Davari M, Karyani AK (2021). Eliciting the public preferences for pharmaceutical subsidy in Iran: a discrete choice experiment study. J Pharm Policy Pract.

