# Supplementary material for: Eliciting the public preferences for pharmaceutical subsidy in Iran: a discrete choice experiment study
Source: J Pharm Policy Pract. 2021 Jul 13;14:59. doi: 10.1186/s40545-021-00345-4 (PMC8278681; doi:10.1186/s40545-021-00345-4)
Supplement: Supplementary file 1 — Additional file 1. Methodology of identification of attributes (literature systematic review and interview with experts). [file 40545_2021_345_MOESM1_ESM.pdf]

2. Methodology

2.1. First step (Identification of attributes)

In the first step for identifying the attributes, a systematic review and interviews with experts were conducted**.**

2.1.1. First step (Literature systematic review)

In the first step, a literature was searched systematically using the following databases: PubMed, Embase, Scopus, and Web of Science. In addition, Google and Google Scholar were searched to identify any non-indexed published or gray literature. Our search strategy was based on key terms: Subsid*, Preference*, and Drug*. The main search key terms were combined using the Boolean operator ‘AND’, and the keywords of the same domain were combined using the Boolean operator ‘OR.’ we did not apply time limits in our search, thereby, we covered all results before October 2018. The search strategy in different databases was as illustrated in (Table 1).

TABLE 1. THE SEARCH STRATEGY IN DIFFERENT DATABASES

Database	Search strategy
**PubMed**	(((((Fund*[Title/Abstract]) OR “Resource Allocation”[Title/Abstract]) OR Subsid*[Title/Abstract]) OR “Priority Setting”[Title/Abstract]) OR Reimbursement*[Title/Abstract])))) AND (((Preference*[Title/Abstract] OR "community view*"[Title/Abstract] OR "social view*"[Title/Abstract] OR "social value*"[Title/Abstract] OR "decision making"[Title/Abstract] OR "societal view*"[Title/Abstract] OR "societal value*"[Title/Abstract] OR "public view*"[Title/Abstract] OR "public value*"[Title/Abstract])))) AND (((drug*[Title/Abstract] OR medicine*[Title/Abstract] OR medication*[Title/Abstract] OR pharma*[Title/Abstract])))
**SCOPUS**	((TITLE-ABS-KEY (fund*) OR TITLE-ABS-KEY ("Resourse Allocation") OR TITLE-ABS-KEY (subsid*) OR TITLE-ABS-KEY ("Priority Setting") OR TITLE-ABS-KEY (reimbursement*))) AND ((TITLE-ABS-KEY (preference*) OR TITLE-ABS-KEY ("community view*") OR TITLE-ABS-KEY ("social view*") OR TITLE-ABS-KEY ("decision making") OR TITLE-ABS-KEY ("social value*") OR TITLE-ABS-KEY ("societal view*") OR TITLE-ABS-KEY ("public view*") OR TITLE-ABS-KEY ("societal value*") OR TITLE-ABS-KEY ("public value*"))) AND ((TITLE-ABS-KEY (drug*) OR TITLE-ABS-KEY (medicine*) OR TITLE-ABS-KEY (medication*) OR TITLE-ABS-KEY (pharma*)))
**Web of Science**	(TS=(subsid*) OR TS=("Priority Setting") OR TS=(reimbursement*) OR TS=("Resource Allocation") OR TS=(fund*)) AND (TS=(preference*) OR TS=("community view*") OR TS=("social view*") OR TS=("decision making") OR TS=("social value*") OR TS=("societal view*") OR TS=("public view*") OR TS=("societal value*") OR TS=("public value*")) AND (TS=(drug*) OR TS=(medicine*) OR TS=(medication*) OR TS=(pharma*))
**EMBASE**	('fund*':ab,ti OR 'priority setting':ab,ti OR 'resourse allocation':ab,ti OR 'subsid*':ab,ti OR 'reimbursement*':ab,ti) AND (preference*:ab,ti OR 'community view*':ab,ti OR 'social view*':ab,ti OR 'social value*':ab,ti OR 'decision making':ab,ti OR 'societal view*':ab,ti OR 'societal value*':ab,ti OR 'public view*':ab,ti OR 'public value*':ab,ti) AND ('pharma*':ab,ti OR 'drug*':ab,ti OR 'medication*':ab,ti OR 'medicine*':ab,ti)

2.1.1.1. Eligibility criteria

Inclusion criteria encompass the following:

1- Studies on the main subject of research and examine the factors influencing the decisions on allocating resources to drugs by evaluating the revealed and stated preferences of policymakers, payers, providers and the people;

2- Studies on resources allocation that addressed only medicines;

3- The study design which includes (conjoint analysis, multi-criteria decision analysis, etc.) and qualitative methods (interviews, focus group discussion, expert panel);

4- The studies which were published in English in peer-reviewed journals;

5- Studies which have a complete structure;

6- Studies which were of primary type and case studies;

7- Studies with available full-text.

2.1.1.2. Exclusion criteria

Studies were excluded when:

1- They were not related to the main research question;

2- They were related to resources allocation that have dealt with other health technologies other than medicines;

3- Their full text is not available;

4- They are of low quality;

4- There is a sort of repetition;

5- There is an updated version of the study;

6- The study is not of primary type such as reviews and editorials.

2.1.1.3. Data extraction

Data from the included studies were extracted by two independent researchers using a predesigned data extraction tool. The extracted data included the following: authors, date of publication, country, study purpose, methodology, sample size, and study perspective. All the extracted data were reported descriptively using diagram following the Preferred Reporting Items for Systematic Reviews and Meta-Analyses (PRISMA) [1].

2.1.1.4. Quality assessment

The quality of the studies was independently assessed by two researchers using the quality assessment tool designed by Kmetet al.[2]. This instrument is composed of two quality assessment checklists with 14 and 10 criteria for quantitative and qualitative studies, respectively, on a 3-point Likert scale (yes=2, partial=1; no=0). All studies which have met 70% of the 14 quantitative or 10 qualitative criteria were included.

2.1.1.5 Data analysis

Extracted studies in each of the databases were entered into the EndNote software, and duplicates were removed. Studies were reviewed in the following steps: 1- Removal of duplicates by EndNote 2- Filtering studies through their titles 3- Evaluation of the remaining studies by quality assessment tool designed by Kmetet et al. 4- Evaluation of the selected studies by two independent researchers 5- Entry of the eligible studies by two independent researchers.

2.1.2. First step (Interviews with experts)

In the first step, also, the opinions of experts (key informants) were obtained using a semi-structured interview guide. Interviewees were selected using the purposive and snowball sampling techniques up to saturation [3]. Initially, a list of experts from some organizations related to the

research context was prepared. By interviewing those experts In the initial list, some other experts were introduced and added to the list (i.e. snowball sampling) [4].
The stakeholders of the drug subsidy included in four main categories: parliament, government organizations, NGOs, and the pharmaceutical industry. Therefore, interviews were conducted with health policymakers familiar with pharmaceutical policies and regulatory in the parliament, governmental organizations, and NGOs. Interviews were also conducted with pharmaceutical experts at the Food and Drug Agency, Ministry of Health and Medical Education, and insurance companies, Managers of pharmaceutical companies, and faculty members who work as advisors for pharmaceutical companies. Therefore, a total of 51 experts in the challenges of drug subsidy or funding were involved in these interviews. Necessary coordination was made beforehand, and invitations were sent. Prior to these interviews, the study objectives were explained by phone calls or face-to-face conversations to the interviewees.
The interview guide included interviewees' demographic characteristics and items related to the study objectives. The following issues were investigated during the interviews:
1- What are the challenges related to allocating subsidies to medicines in the country? What solutions do you recommend to achieve the desired situation?
2- What are the current criteria for allocating medicine subsidies in the country?
3- Do we have similar criteria for the entry of medicines to the list of subsidized medicines, including those related to, incurable and chronic diseases, infertility, etc.? Explain.
4- Do you think it is necessary to make changes in the current criteria for including a particular medicine in the list of subsidized medicines as well as the level of subsidies? What criteria should be considered? What is the rate (percentage) for each drug?
In addition to audio recording, field notes were also taken for more accuracy of data collection. After transcribing the interviews, confidentiality was preserved by hiding the personal information. After completing the interviews, important attributes of allocate subsidies to medicines were extracted and listed. All interviews took place in the interviewees’ workplaces. Data collection and anlysis were conducted between October 2018 and June 2019.
Therefore, a list of attributes from the systematic review and interviews with experts was developed.

2.2. Second step (Determine the final attributes and levels)

There were some limitations in the number of attributes and levels, which was included in the final design of the DCEs study [5, 6]. Accordingly, this was aimed to determine the final attributes and levels of subsidy allocation to a drug. Therefore, in the second step, the first research team meeting was carried out in order to examine the identified attributes. Team members in this meeting excluded similar attributes and discussed each attribute thoroughly aiming at reaching agreement on a shortlist of attributes.
According to recent reviews, it has been reported that most DCEs used a number of attributes between 4 and 7 [5, 7]. Therefore, in this step, a panel comprised of five experts discussed the aforementioned attributes [8]. At the beginning of that meeting, the panel members were asked to select from aforementioned attributes in the initial set, thus, seven important attributes have been highlighted based on the need of policymakers to consider in allocating subsidies to drugs in the Iranian health system.
In each round, every member in the meeting has discussed one of the seven important attributes, and if more than three experts agreed on that, it was approved, and the next round of the meeting discussed the next important attribute. The first expert in the panel started with suggesting the first

attribute for discussion, and the first round ended when another expert suggested the next important attribute, and so on until the fifth expert.

Selecting experts for the panel was purposive. We included those experts with at least 3 years of work experience related to drug reimbursement decisions or the Supreme Council of Health Insurance and basic insurance package or Iran Drug List Compilation Council or the Pharmaceutical Memorandum Commission. One expert was from the field of economics and pharmaceutical management involved in previous drug subsidy decisions. One was a pharmacist and had previously participated in making decisions to compile a basic insurance package. One was an expert in health economics, one was experienced in economics and pharmaceutical management, and one has a sufficient experience in health policy. They were all invited to take part via telephon where the purpose and the study context were explained. They expressed their willingness to participate in view of the importance of subsidy in improvement of patients' access to medicines and reducing their out-of-pocket payment.

3. Results

3.1. First step (Identification of attributes)

In the first step, attributes have been identified through a systematic review and the interviews with experts**.** In this line, a list of different attributes for allocating subsidy and resources to a drug was developed, both in Iran and other countries.

3.1.1. First step (Literature systematic search)

The literature was systematically searched and a total of 11770 abstract citations; 1944 from PubMed, 1834 from Embase, 586 from Scopus, 2131 from Web of Science, and one was added manually from abstracts Google. After removing 4017 duplicates, 7754 titles and abstracts were screened for eligibility, of which, only 59 abstracts were found to be potentially eligible. The eligible abstracts were moved to the next step for full-text screening. Nine of these full-text studies were excluded, while 50 records were eligible. A total of 50 articles were included for the final review (Figure 1). After completing the systematic review, important attributes of resource allocation to medicines were extracted and listed. The systematic literature review revealed a number of 124 attributes.

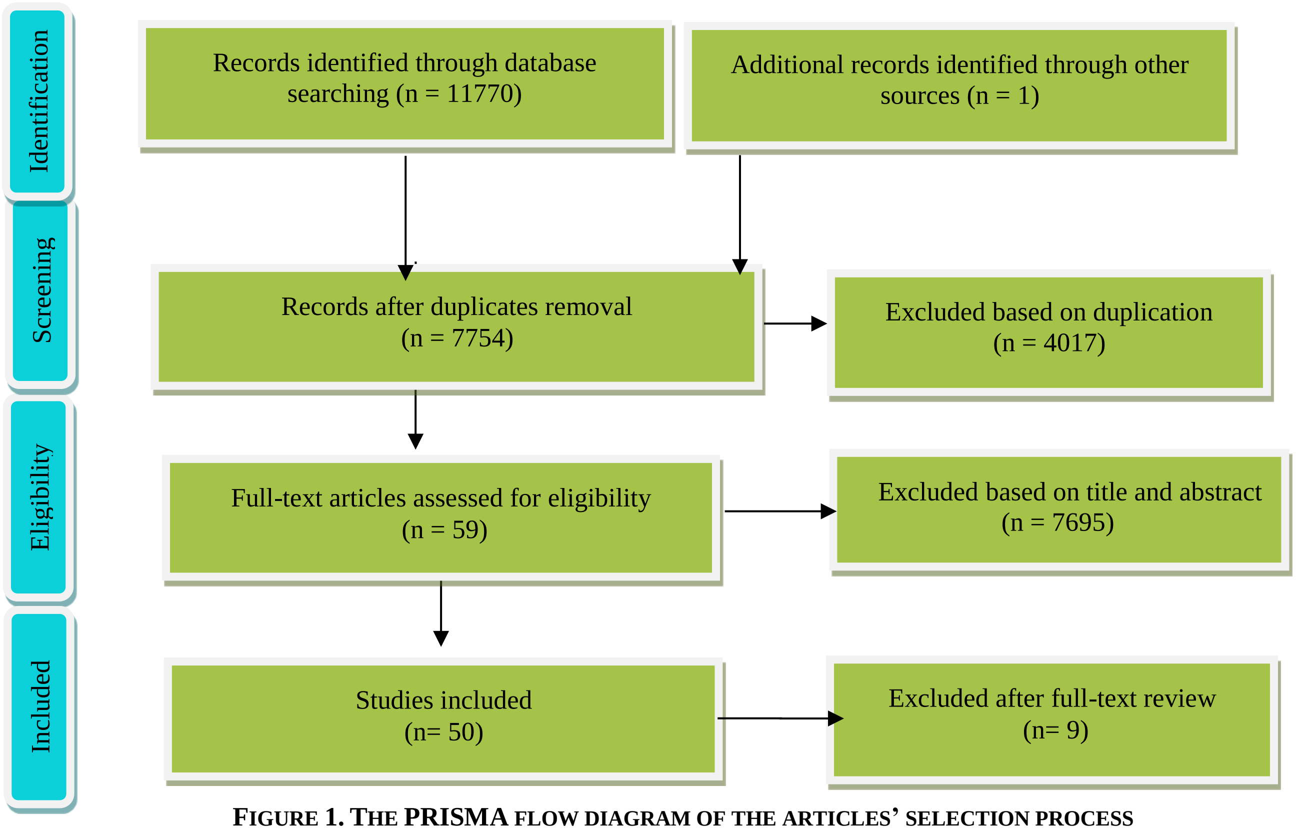

FIGURE 1. THE PRISMA FLOW DIAGRAM OF THE ARTICLES' SELECTION PROCESS

3.1.1.1 Studies characteristics

Although we have not applied time limits to our search, all 50 included articles were published after 2000 (Table 2). About one third (34%) and two thirds (66%) of the studies were published during the last 3 years and 6 years, respectively. Most studies were conducted in the United Kingdom (12 studies), Australia (12 studies) and Canada (10 studies). Most of the included studies (33 studies) examined the revealed preferences of policymakers. Twenty four studies also examined the stateded preferences of policymakers and the public. Most of the studies were conducted of the policymakers' perspective (41 studies). 13 studies were conducted from the perspective of the public and 2 studies were conducted from the perspective of health care providers. In most of the studies (33 studies) policy documents related to drug decisions were reviewed. The highest sample size in the study of revealed and stated preferences were 977 drug decisions and 4118 people, respectively. The most common methods for selecting attributes in these studies included review of published policy documents, review of literature, focus group discussion (FGD), and interviews with experts. The minimum and maximum number of attributes used in these studies were 1 attribute (9 studies) and 14 attributes (1 study), respectively, with the highest repetition being related to 1 attribute (9 studies) and 7 attributes (6 studies). Therefore, after completing the systematic review, important attributes of allocating resources to medicines were extracted and listed.

3.1.2. First step (Interview with experts)

Then, after interviewing the experts who were listed in the initial list, some interviewees were introduced by experts (i.e. snowball sampling). A total of 51 semi-structured interviews were conducted, with a wide range of participants including parliament members, experts of the Planning and Budget Organization, Secretariat of the Supreme Insurance Council, Ministry of Health, Medical Universities, Food and Drug Administration, Health Insurance Organizations, Transparency and Justice Watch Organization, Pharmaceutical Industry, and Charity Foundation for Special Diseases. Fourteen (out of 51) participants have not mentioned an attribute for allocating subsidies to medicines. The lowest and highest attributes mentioned in an interview were one and 10, respectively. The most frequently mentioned attribute was cost-effectiveness (i.e. 15). The least frequent attributes were physicians' demands, paying attention to social perspective to disease, independence of committee on the evaluation of allocation of medicine subsidies, having a family member with a chronic disease, having a history of receiving a drug that is on the list of subsidized, insurance coverage, and history of investigating the drug at the Joint Committee of the Food and Drug Administration and health insurance funds (Tables 2). The Interviewees with key informants revealed a number of 64 attributes.

TABLE 2. LIST OF CHARACTERISTICS OF THE PARTICIPATING PEOPLE AND ORGANIZATIONS IN THE PRESENT STUDY AND EXTRACTED ATTRIBUTES

Organizational affiliation	Description	Number	Sum	Extracted Attributes (according to interviews)
**Parliament (health commission)**	Physician (Ear, nose, and throat specialist)	1	4	1- Expensiveness of medicine<br>2- The effect of the chronic nature of the disease on the duration of need for medication<br>3- Based on income deciles<br>4- Catastrophic costs of treatment
	Nurse (Ph.D. in nursing)	1		
	Physician (Hematologist)	1		
	Physician (Ophthalmologist)	1		
**Planning and Budget Organization**	Physician (Ph.D. in health policy)	1	3	1- Quality-Adjusted Life Years
	Physician (Ph.D. in Physiology)	1		
	Expert	1		
**Ministry of health (deputy for treatment affairs)**	Physician (radiotherapist)	1	5	1- Lifelong disease<br>2- Duration of the disease<br>3- Imposed financial burden for the patient<br>4- Total number of those who suffer from the disease<br>5- Catastrophic costs of treatment<br>6- Externalities of the target medicine<br>7- Severity of the disease<br>8- Physicians' demands<br>9- Domestic production<br>10- Cost-effectiveness of the drug<br>11- High-quality life expectancy<br>12- Age groups of patients
	Physician (Ph.D. in Nutrition)	1		
	Physician (International Health Management Course in the UK)	1		
	Physician (Ph.D. in Social Medicine)	1		
	Physician	1		
**Ministry of Welfare**	Pharmacist	1	1	1- Lack of alternative treatment<br>2- Independence of committee on the evaluation of allocating subsidies to medicines
**Food and Drug Administration**	Pharmacist (Ph.D. in Pharmaceutics)	1	6	1- Imposed cost of the disease<br>2- Outbreak of the disease<br>3- Rational prescription<br>4 -Cost-effectiveness<br>5- Not allocating subsidy to diseases that are due to lifestyle changes<br>6- Availability of alternative medicine<br>7- Reimbursement<br>8- Treatment period<br>9 -Number of patients who receive the medicine<br>10 -Price of medicine<br>11- Economic status of patients<br>12- Expensive of the medicine<br>13- Rarity of the disease<br>14- Quality of Life
	Pharmacist (Ph.D. in Pharmaceutical Economics and Management)	4		
	Pharmacist (Ph.D. in Pharmacology)	1		

				15- Duration of drug use due to the chronic nature of the disease<br>16- Externalities<br>17- Severity of the disease<br>18- Budget Impact<br>19- Prioritization of children, due to the training of the country's future skilled workers<br>20- Ability to buy medicines<br>21- The highest productivity for most of the people<br>22- Disability-adjusted life years<br>23- Indicators of equity
**Pharmaceutical industry (syndicate of manufacturers, and importers, distributors, and distributors of medicines)**	Pharmacist<br>Chemical engineering<br>Pharmacist (Ph.D. in Pharmacology)<br>Pharmacist (Ph.D. in Physiology)	5<br>1<br>2<br>1	9	1- QALY<br>2- DALY<br>3- Cost-effectiveness<br>4- Age group of patients<br>5- Prioritizing the productive workforce of the society<br>6- Domestic Production<br>7- Burden of disease (incidence and prevalence)<br>8- Number of patients<br>9- Chronicity of the disease<br>10- Budget Impact<br>11- More coverage of the elderly<br>12- More coverage of pregnant women<br>13- Prioritizing costly drugs<br>14- Prioritizing patients with lower financial ability<br>15- Type of the disease<br>16- Stage of the disease (i.e. prescription for end-stage patients or at the chronic stage)<br>17- Alternative medicines<br>18- Availability of domestically produced drugs<br>19- Effectiveness<br>20- Price of medicine<br>21- Income Deciles<br>22- Long-term consumption<br>23- The ratio of medicine expenditures to the person's income
**Health insurance organizations** / **Health insurance organization**	Physician (ophthalmologist)<br>Physician (Ph.D. in Epidemiology)<br>Pharmacist<br>Physician (Ph.D. in Health Policy)	1<br>1<br>2<br>1	5	1- Catastrophic costs of treatment<br>2- Cost-effectiveness<br>3- Effectiveness<br>4- High costs (both price and frequency of consumption)<br>5- Economic status of patients<br>6- Domestic production<br>7- Low cost alternatives<br>8- Not prioritizing diseases that are due to lifestyle changes<br>9- Age group of patients

					(prioritizing favoring young over old)
	**Social security organization**	Pharmacist	1	4	1- Difficulty or incurability of the disease<br>2- Prevalence of the disease<br>3- Rising incidence<br>4- Current expenditures for medicines and other treatments<br>5- The importance of the disease<br>6- Expensiveness of the medicine<br>7- Treatment Period<br>8- Having a history of receiving a drug that is on the list of prescribed medicine<br>9- History of investigating the drug at the Joint Committee of the Food and Drug Administration and health insurance funds<br>10- Cost-effectiveness<br>11- Domestic production<br>12- Age groups of patients (prioritizing children to the elderly)<br>13- Effectiveness<br>14- Type of the disease (having externality)<br>15- High ratio of drug price to patients' income<br>16- Having a family member with a chronic disease
		Physician	2		
		Physician (Ph.D. in Health Economist)	1		
	**Imam Khomeini Relief Foundation (merged with the Iran health insurance organization)**	Physician	1	3	1- Similar medicine with a higher price<br>2- Rational use<br>3- Cost-effectiveness<br>4- Patients' needs<br>5- Categorization of patients<br>6- Failure to create an induction market
		Nurse	1		
		Pharmacist	1		
	**Armed Forces Medical Services Insurance Organization**	Pharmacist	1	1	1- Cost-effectiveness<br>2- Doesmtic production
**Medical Universities**		Physician (Ph.D. in pharmacology)	1	4	1- Studying the economic burden through marginal analysis of drug effectiveness<br>2- Number of patients<br>3- QALY<br>4- Effectiveness<br>5- Determination of income deciles<br>6- DALY<br>7- Cost-effectiveness<br>8- Budget Impact<br>9- Reasonable price
		Pharmacist (Ph.D. in Pharmacology)	1		
		Pharmacist (Ph.D. in Health Economist)	1		
		Pharmacist (Ph.D. in Pharmaceutical Economics and Management)	1		

				10- Safety<br>11- Severity of disease<br>12- Budget Impact<br>13- Prioritizing children, pregnant women, children, and adolescents<br>14- Fair access to medicine<br>15- Impoverishment due to health expenditures<br>16- Emphasizing equity and transparent philanthropy<br>17- Affected population<br>18- Prioritizing drugs that their demand is not influenced by induction
**Organizational on Transparency and Justice Watch**	Expert	1	1	1- Prioritizing medications that do not induce demand
**Support Associations**	Physician (neurologist and MS fellowship)	1	3	1- Paying attention to social perspective to disease
	Pharmacist	1		
	Law Expert	1		
**Cancer research center**	Physician (cancer surgeon)	1	1	1- Overall survival (relative to free survival)<br>2- Improving the patients' quality of life<br>3- Cost-benefits<br>4- Equity
**Tehran Chamber of Commerce**	Pharmacist	1	1	No
**Total**	51			

3.2. Second step (Determine the final attributes and levels)
In the second step**,** research team meeting was carried out to examine the identified attributes, which in turn yielded 34 attributes (Table 3).
Furthermore, in this step, the attributes have been finalized by the expert panel. Hence, a panel comprised of five experts discussed the aforementioned attributes. With holding 12-round sessions, a list of seven attributes were higlighted, encompassing: increasing survival after treatment, promoting quality of life (QoL) after treatment, alternative treatment, age group of the target population, cost burden for the government, disease severity, and medicine-manufacturing country. Also, the appropriate number of levels for each attribute was determined (Table 4).

TABLE 3. DETERMINED ATTRIBUTES FROM THE LITERATURE SYSTEMATIC REVIEWS AND INTERVIEW WITH EXPERTS FOR ALLOCATING SUBSIDIES TO MEDICINES

#	Determined attributes for allocating subsidies to medicines
1	Effectiveness of medicine
2	Safety of medicine
3	Domestic production (generic) of medicine
4	Price of medicine
5	Similar drug with a higher price
6	Availability of alternative options
7	Cost-effectiveness of medicine
8	Certainty of cost-effectiveness of medicine
9	Enhancing the quality of life of medicine
10	Increasing life expectancy of medicine
11	Incremental cost-effectiveness ratio of medicine
12	Reimbursement status or allocation of subsidy to medicine in other countries, especially those with a similar context to Iran
13	Treatment protocol (inclusion of the drug in the first or second line of the treatment)
14	Possibility to prescribe the drug for several indications
15	Not prioritizing diseases that are due to lifestyle changes
16	The age group of the target population, in terms of productiveness (productivity) and vulnerability
17	Number of patients (prevalent or rare)
18	The burden of disease (incidence and prevalence)
19	Severity of disease
20	The probability (chance) of treatment success
21	Socioeconomic status of the target group (considering the household's income instead of sole attention to the income of the patient him/herself)
22	Catastrophic costs of treatment
23	The budget impact (share of the target drug from the total subsidies allocated to the medicines)
24	History of investigating the drug at the Joint Committee of the Food and Drug Administration and health insurance funds;
25	Having a history of receiving a drug that is on the list of the prescribed drugs
26	Physicians' demand
27	Considering a social perspective to disease (being a subsidized drug, for drugs related to diseases with a high social sensitivity (e.g. thalassemia patients who have less job opportunities compared to those who suffer from other chronic diseases)
28	Extensive social benefits (positive externalities)
29	Drugs developed for end stage patients
30	Cancer related drugs
31	Innovation in production
32	For drugs are prone to inducing demand, not receiving the subsidy
33	Equitable access
34	Independence of committee on the evaluation of allocating subsidies to medicines

TABLE 4. ATTRIBUTES AND LEVELS USED IN THE DCE

Attribute	Definition	Level
		**Pharmaceuticals A, B**
**Increasing survival after treatment**	The average number of years increased to the patients' life by taking the drug.	No effect on the patients' longevity (remaining in the previous lifetime)
		Low: 1 year
		Average: 5 year
		High: 10 year
**promoting quality of life after treatment**	Improved health-related quality of life due to consuming a drug. According to the World Health Organization, four dimensions of health are physical, mental, social, and spiritual. Therefore, in this study, we only considered the health-related quality of life.	No effect on QoL of patients (previous QoL)
		Low improvement in QoL (15%)
		Average improvement in QoL (30%)
		High improvement in QoL (50%)
**Alternative treatment**	Other treatments such as surgery, radiotherapy, and other drugs with similar mechanisms of action.	Yes
		No
**Age group of the target population**	The age range of those who consume the drug.	Less than 18 years of age
		18 to 60y
		Over 60y
		All age groups
**Cost burden for the government**	The annual budget that the government allocates as subsidy for medicines.	Low: 10 million IRR (≈240 US dollar)
		Average: 100 million IRR (≈2380 US dollar)
		High: 500 million IRR (≈11900 US dollar)
**Disease severity**	Patients' longevity and QoL before the onset of drug use.	Mild: high longevity (15 years), moderate QoL [60%]
		Moderate: High longevity (15 years), low QoL (30%)
		Severe: low longevity (up to 3 month) low QoL (30%)
**Drug manufacturer country**	The final product is produced in Iran or is imported.	Domestic production
		Imported

Sampling

The sampling method was designed based on differences between 22 districts of Tehran city concerning geographical distribution of economic, social, spatial, cultural, and recreational indicators. Based on a descriptive-analytical study [9] that used the factor analysis model, cluster analysis, human development index, and Williamson scattering coefficient to categorize the 22 regions of Tehran according to economic, social, spatial, cultural, and recreational indicators into three groups of developed, semi-developed, and under-developed, six regions (out of 22) were selected. The sample size was calculated as 408 households, which was multiplied by three (i.e. 1224). Therefore, two regions were randomly selected from each area and 408 households were interviewed from each region, proportionate to their population share (Table 5).

Table 2. Categorization of 22 regions of the city of Tehran based on economic, social, spatial, cultural, and recreational indicators

**Highly Developed regions (five districts)**	**Semi-developed regions (four districts)**	**Less developed regions (13 districts)**
1, 2, 3, 6, 7	4, 5, 11, 13	8, 9, 10, 12, 14, 15, 16, 17, 18, 19, 20, 21, 22
Random Selection		
1 and 6	4 and 11	14 and 17
The total sample size was determined as 1224 households (408 from each region). The number of households was determined based on the population share of each region.		

References

1. Moher D, Liberati A, Tetzlaff J, et al. Preferred reporting items for systematic reviews and meta analyses: the PRISMA statement. PLoS Med., 2009 Jul. 21;6(7):e1000097.

2. Kmet LM, Cook LS, Lee RC. Standard quality assessment criteria for evaluating primary research papers from a variety of fields. Alberta Heritage Foundation for Medical Research (AHFMR);. 2004. http://www.ihe.ca/documents/HTA-FR 14.pdf

3. Hanson JL, BD, Giardino AP, Qualitative research methods for medical educators. Academic Pediatrics. , 2011. 11(5):375–86.

4. Green J, Thorogood N. Qualitative methods for health research. sage; 2018 Feb 26.

5. de Bekker-Grob EW, Ryan M, Gerard K. Discrete choice experiments in health economics: a review of the literature. Health economics. 2012 Feb;21(2):145-72.

6. Ryan M, Kolstad JR, Rockers PC, Dolea C. How to conduct a discrete choice experiment for health workforce recruitment and retention in remote and rural areas: a user guide with case studies. The World Bank; 2012 Dec 20.

7. Marshall D, Bridges JF, Hauber B, Cameron R, Donnalley L, Fyie K, Johnson FR. Conjoint analysis applications in health—how are studies being designed and reported?. The Patient: Patient-Centered Outcomes Research. 2010 Dec;3(4):249-56.

8. Humphrey-Murto S, Varpio L, Wood TJ, Gonsalves C, Ufholz LA, Mascioli K, Wang C, Foth T. The use of the Delphi and other consensus group methods in medical education research: a review. Academic Medicine. 2017 Oct 1;92(10):1491-8.

9. Mirzaei J, AS. Lorestani A, Mirzaei J. Spatial Analysis of Prosperity Levels in Tehran Metropolis from the Perspective of Urban Economics. Journal of Urban Economics and Management. 2015 Sep 10; 3(11):59-77.
