# Supplementary material for: Eliciting the public preferences for pharmaceutical subsidy in Iran: a discrete choice experiment study
Source: J Pharm Policy Pract. 2021 Jul 13;14:59. doi: 10.1186/s40545-021-00345-4 (PMC8278681; doi:10.1186/s40545-021-00345-4)
Supplement: Supplementary file 2 — Additional file 2. Questionnaire of public preferences evaluation. [file 40545_2021_345_MOESM2_ESM.pdf]

Questionnaire on evaluation of preferences towards* allocating subsidies *to* medicines *in the Iranian health system

Dear participant,
We are kindly looking for your participation in this study entitled: "Allocating Subsidies to Medicines in the Iranian Health System: Policy Analysis and Framework Development by Emphasis on Specific and Incurable Diseases". A total of 1 224 households will take part in this study and you have been selected as the head or member of the household. First, we are going to provide you a brief explanation about medicine subsidies in Iran.
"The Government of the Islamic Republic of Iran allocates annual funds to reduce the price of medicines, which is mainly focused on medicines related to special and incurable diseases. These subsidies are mainly financed through general taxes, targeted subsidies plan, and selling natural resources such as oil and gas, etc. Due to budget limitations, subsidies cannot be allocated to all medicines, hence, there should be a sort of tradeoff. Based on what was mentioned before, you think what medicines are eligible for subsidization?"
The current questionnaire containing three parts intends to evaluate your preferences for the allocation of medicine subsidies. The first part is about the choice of drugs for subsidy allocation according to their characteristics. The second part contains sociodemographic information such as age, gender, education, occupational status, and type of insurance. Finally, the third part is about your current health status.
Your participation is voluntary and the privacy of your information is ensured. If you are interested in the findings, 6 months after filling this questionnaire, please contact us at 09125016542 or you can send an email to Delpasandmansoor@gmail.com. You can also send your enquiries if required.
Your detailed answers to the questions can be used for the development of a guide for managers and policymakers to optimize resource allocation and targeting subsidies in the Iranian healthcare system (IHS). The research team acknowledges your sincere cooperation.
Note: the respondent should be the head of the family with a diploma degree, at least, or a family member with at least 18 years old.

Interviewers' name:
Date:
Questionnaire ID:
Questionnaire Code:

Part A: This section contains 8 items with two different types of drugs (A and B). These two medicines can be used for various diseases. Each of A and B drugs has seven different characteristics. Please, carefully read the definitions of all seven characteristics, and then choose one of the provided drugs.
Note that there is no true or false answer.

**Pharmaceutical A**	Question 1	**Pharmaceutical B**
Severe= low longevity (up to 3 month) low QoL (30%)	**Disease severity**	Mild= high longevity (15 years), moderate QoL (60%)
No	**Alternative treatment**	Yes
10 years	**Survival**	5 years
Average improvement in QoL (30%)	**Quality of life**	Low improvement in QoL (15%)
10 million IRR (240 US dollar)	**Cost to government**	500 million IRR (11900 US dollar)
Domestic production	**Drug manufacturer country**	Domestic production
All age groups	**Age group**	All age groups
Which pharmaceutical do you prefer to be subsidized? **Pharmaceutical A** ☐ **Pharmaceutical B** ☐		

**Pharmaceutical A**	Question 2	**Pharmaceutical B**
Mild= high longevity (15 years), moderate QoL (60%)	**Disease severity**	Moderate= High longevity (15 years), low QoL (30%)
Yes	**Alternative treatment**	No
1 year	**Survival**	5 years
High improvement in QoL (50%)	**Quality of life**	No effect on QoL of patients (previous QoL)
10 million IRR (240 US dollar)	**Cost to government**	500 million IRR (11900 US dollar)
Domestic production	**Drug manufacturer country**	Imported
Less than 18 years of age	**Age group**	Over 60y
Which pharmaceutical do you prefer to be subsidized? **Pharmaceutical A** ☐ **Pharmaceutical B** ☐		

**Pharmaceutical A**	Question 3	**Pharmaceutical B**
Mild= high longevity (15 years), moderate QoL (60%)	**Disease severity**	Severe= low longevity (up to 3 month) low QoL (30%)
No	**Alternative treatment**	Yes
10 years	**Survival**	No effect on the patients' longevity (remaining in the previous lifetime)
Average improvement in QoL (30%)	**Quality of life**	High improvement in QoL (50%)
500 million IRR (11900 US dollar)	**Cost to government**	10 million IRR (240 US dollar)
Imported	**Drug manufacturer country**	Domestic production
All age groups	**Age group**	Over 60y
Which pharmaceutical do you prefer to be subsidized? **Pharmaceutical A** ☐ **Pharmaceutical B** ☐		

**Pharmaceutical A**	Question 4	**Pharmaceutical B**
Moderate= High longevity (15 years), low QoL (30%)	**Disease severity**	Severe= low longevity (up to 3 month) low QoL (30%)
Yes	**Alternative treatment**	No
1 year	**Survival**	10 years
No effect on QoL of patients (previous QoL)	**Quality of life**	Low improvement in QoL (15%)
10 million IRR (240 US dollar)	**Cost to government**	500 million IRR (11900 US dollar)
Domestic production	**Drug manufacturer country**	Imported
Less than 18 years of age	**Age group**	All age groups
Which pharmaceutical do you prefer to be subsidized? **Pharmaceutical A** ☐ **Pharmaceutical B** ☐		

**Pharmaceutical A**	Question 5	**Pharmaceutical B**
Severe= low longevity (up to 3 month) low QoL (30%)	**Disease severity**	Mild= high longevity (15 years), moderate QoL (60%)
No	**Alternative treatment**	Yes
1 year	**Survival**	5 years
No effect on QoL of patients (previous QoL)	**Quality of life**	Low improvement in QoL (15%)
500 million IRR (11900 US dollar)	**Cost to government**	100 million IRR (2380 US dollar)
Imported	**Drug manufacturer country**	Domestic production
18 to 60y	**Age group**	All age groups
Which pharmaceutical do you prefer to be subsidized? **Pharmaceutical A** ☐ **Pharmaceutical B** ☐		

**Pharmaceutical A**	Question 6	**Pharmaceutical B**
Moderate= High longevity (15 years), low QoL (30%)	**Disease severity**	Severe= low longevity (up to 3 month) low QoL (30%)
Yes	**Alternative treatment**	No
10 years	**Survival**	5 years
High improvement in QoL (50%)	**Quality of life**	No effect on QoL of patients (previous QoL)
500 million IRR (11900 US dollar)	**Cost to government**	100 million IRR (2380 US dollar)
Imported	**Drug manufacturer country**	Imported
Over 60y	**Age group**	Less than 18 years of age
Which pharmaceutical do you prefer to be subsidized? **Pharmaceutical A** ☐ **Pharmaceutical B** ☐		

**Pharmaceutical A**	Question 7	**Pharmaceutical B**
Severe= low longevity (up to 3 month) low QoL (30%)	**Disease severity**	Moderate= High longevity (15 years), low QoL (30%)
Yes	**Alternative treatment**	No
1 year	**Survival**	10 years
Average improvement in QoL (30%)	**Quality of life**	High improvement in QoL (50%)
500 million IRR (11900 US dollar)	**Cost to government**	10 million IRR (240 US dollar)
Domestic production	**Drug manufacturer country**	Domestic production
Over 60y	**Age group**	18 to 60y
Which pharmaceutical do you prefer to be subsidized? **Pharmaceutical A** ☐ **Pharmaceutical B** ☐		

**Pharmaceutical A**	Question 8	**Pharmaceutical B**
Moderate= High longevity (15 years), low QoL (30%)	**Disease severity**	Mild= high longevity (15 years), moderate QoL (60%)
Yes	**Alternative treatment**	No
No effect on the patients' longevity (remaining in the previous lifetime)	**Survival**	1 year
Low improvement in QoL (15%)	**Quality of life**	Average improvement in QoL (30%)
500 million IRR (11900 US dollar)	**Cost to government**	10 million IRR (240 US dollar)
Imported	**Drug manufacturer country**	Domestic production
18 to 60y	**Age group**	Over 60y
Which pharmaceutical do you prefer to be subsidized? **Pharmaceutical A** ☐ **Pharmaceutical B** ☐		

Part 2: Personal Characteristics	
1	Personal Characteristics: 1) Male 2) Female
2	Data of Birth …..
3	Marriage Status: 1) Married 2) Single due to divorce, death of the partner 3) Never married
4	Are you the head of your family? 1) Yes 2) No<br>If answered no, please describe your position in the family ……
5	Number of family members: …..
6	Education Level: Tertiary Education 2) Associate 3) Bachelor 4) Master 5) Doctoral 6)Religious education
7	A) Have you been covered by health insurance for the past year? 1) Yes 2) No<br>B) If yes, please select your insurer:<br>1) Social Security Insurance 2) Iranian Healthcare Insurance Organization 3) Armed Forces Health Insurance 4) Imam Khomeini Relief Foundation 5) Others.
8	A) Have you been covered by complementary health insurance for the past year? 1) Yes 2) No<br>B) If yes, please mention the name of your insurer
9	Occupation status: 1) Public sector 2) Private sector 3) Housekeeper 4) Unemployed or disabled 5) Students 6) Retired and pension 7) others.
10	Please select one of the following items that best fits your family's average income during the past months (IRR)?<br>(income includes salary, received subsidies, and profits gained from investments)<br>1) Up to 10 million IRR; 2) 11 000 000 to 20 000 000; 3) 21 000 000 to 30 000 000; 4) 31 000 000 to 40 000 0000; 5) 41 000 000 to 50 000 000; 6) 51 000 000 to 60 000 000; 7) 61 000 000 to 70 000 000; 8) > 70 000 000.
11	How much you have spent on healthcare services during the past month? …
12	A. Have you been hospitalized over the past year? 1) Yes 2) No<br>b) If yes, how much it costed for you? ....
13	A. Do you have a history of medication over the past month? 1) Yes 2) No<br>b) If yes, how much it costed for you? ....
14	How much your family has paid for healthcare services during the past month? …
15	A. Has one of your family members been hospitalized over the past year? 1) Yes 2) No<br>b) If yes, how much it costed? ....
16	A. Does one of your family members has a history of medication over the past month? 1) Yes 2) No<br>b) If yes, how much it costed? ....
17	Do you some cigarette? 1) Yes 2) No
18	A) Do you suffer from a chronic disease (having a special sickness card or confirmation of the health insurance fund)? 1) Yes 2) No<br>b) If yes, please mention your disease: 1) Hemophilia 2) Thalassemia 3) Dialysis 4) Transplantation (Type of transplantation ……) 5) Cancer (type of cancer ….) 6) MS 7) Butterfly disease 8) Metabolic syndrome (type of the metabolic disease) 9) Other diseases ……..
19	A) If you suffer from one of the above-mentioned diseases, what proportion of your expenditures on medicines can be attributed to these diseases? ....<br>b) please mention the name of medicines that you received
20	A) Does any of your family members suffer from a chronic disease (having a special sickness card or confirmation of the health insurance fund)? 1) Yes 2) No<br>b) If yes, please mention your disease: 1) Hemophilia 2) Thalassemia 3) Dialysis 4) Transplantation (Type of transplantation ……) 5) Cancer (type of cancer ….) 6) MS 7) Butterfly disease 8) Metabolic syndrome (type of the metabolic disease) 9) Other diseases ……..
21	A) If any of your family members suffer from one of the above-mentioned diseases, what proportion of your family's expenditures on medicines can be attributed to these diseases?..............<br>b) please mention the name of medicines that they received
22	Overall, how do you evaluate your health status?<br>1) Excellent 2) Very good 3) Relatively good 4) Relatively bad 5) Quite bad
23	Do you have access to the Internet at your home? 1) Yes 2) No
24	Are you agree with the elimination of subsidies for domestically produced medicines? 1) Yes 2) No
25	Are you agree with the elimination of subsidies for imported medicines? 1) Yes 2) No
26	Do you agree with the removal of monthly cash subsidies of your family in favor of those who suffer from specific and incurable diseases like hemophilia, thalassemia, dialysis, cancer, MS, etc., which their medicines are highly expensive? 1) Yes 2) No

27	Do you agree with the removal of monthly cash subsidies of high-income families in favor of those who suffer from specific and incurable diseases like hemophilia, thalassemia, dialysis, cancer, MS, etc., which their medicines are highly expensive?<br>1) Yes 2) No
28	Do you agree with transferring monthly cash subsidies to a medical saving account to cover the costs of specific and incurable diseases like hemophilia, thalassemia, dialysis, cancer, MS, etc., which their medicines are highly expensive? 1) Yes 2) No
29	Do you agree with the removal of subsidies for Over-the-counter drugs such as acetaminophen in favor of medicines of specific and incurable diseases like hemophilia, thalassemia, dialysis, cancer, MS, etc., which their medicines are highly expensive? 1) Yes 2) No
30	Do you agree with the elimination of subsidies for medicines for specific and incurable diseases?<br>1) Yes 2) No
31	Do you agree with the elimination of subsidies for medicines of specific and incurable diseases for high-income families? 1) Yes 2) No
